# Expression of Estrogen-Related Gene Markers in Breast Cancer Tissue Predicts Aromatase Inhibitor Responsiveness

**DOI:** 10.1371/journal.pone.0077543

**Published:** 2013-11-06

**Authors:** Irene Moy, Zhihong Lin, Alfred W. Rademaker, Scott Reierstad, Seema A. Khan, Serdar E. Bulun

**Affiliations:** 1 Northwestern University, Department of Obstetrics and Gynecology, Chicago, Illinois, United States of America; 2 Northwestern University, Department of Preventive Medicine, Chicago, Illinois, United States of America; 3 Northwestern University, Department of Surgery, Chicago, Illinois, United States of America; Baylor College of Medicine, United States of America

## Abstract

Aromatase inhibitors (AIs) are the most effective class of drugs in the endocrine treatment of breast cancer, with an approximate 50% treatment response rate. Our objective was to determine whether intratumoral expression levels of estrogen-related genes are predictive of AI responsiveness in postmenopausal women with breast cancer. Primary breast carcinomas were obtained from 112 women who received AI therapy after failing adjuvant tamoxifen therapy and developing recurrent breast cancer. Tumor ERα and PR protein expression were analyzed by immunohistochemistry (IHC). Messenger RNA (mRNA) levels of 5 estrogen-related genes–AKR1C3, aromatase, ERα, and 2 estradiol/ERα target genes, BRCA1 and PR–were measured by real-time PCR. Tumor protein and mRNA levels were compared with breast cancer progression rates to determine predictive accuracy. Responsiveness to AI therapy–defined as the combined complete response, partial response, and stable disease rates for at least 6 months–was 51%; rates were 56% in ERα-IHC-positive and 14% in ERα-IHC-negative tumors. Levels of ERα, PR, or BRCA1 mRNA were independently predictive for responsiveness to AI. In cross-validated analyses, a combined measurement of tumor ERα and PR mRNA levels yielded a more superior specificity (36%) and identical sensitivity (96%) to the current clinical practice (ERα/PR-IHC). In patients with ERα/PR-IHC-negative tumors, analysis of mRNA expression revealed either non-significant trends or statistically significant positive predictive values for AI responsiveness. In conclusion, expression levels of estrogen-related mRNAs are predictive for AI responsiveness in postmenopausal women with breast cancer, and mRNA expression analysis may improve patient selection.

## Introduction

Breast cancer is a hormone-dependent disease that relies on the mitogenic effects of estrogen to drive tumorigenesis and tumor growth [1], [2]. The expression of clinically significant levels of estrogen receptor-α (ERα) is seen in approximately 80% of human breast carcinomas whereas the progesterone receptor (PR) is expressed in approximately 55% [3]–[5]. Endocrine therapy is indicated in patients who possess ERα and PR positive tumors. The estrogen pathway and synthesis has been targeted through receptor blockade, reduction in circulating levels of estrogen, or by suppression of synthesis in tissues of women diagnosed with breast cancer [1]–[5].

Aromatase inhibitors (AIs), which selectively inhibit aromatase activity in tissues responsible for estrogen production, have been used for the hormonal treatment of breast cancers. Given the fact that the overwhelming majority of breast cancer is hormone receptor-positive, most women are placed on AI treatment. However, although AIs are very well tolerated with a remarkably low incidence of serious adverse effects, they do carry a risk of osteoporosis and arthralgias [6]. With objective response rates of slightly more than 50% to AI therapy, there is a need for improved predictive methods to better identify patients who will benefit from it and sparing those patients who may not [7].

Here, we attempted to define a molecular signature in breast cancer tissue that can improve the predictive accuracy of a patient’s response to treatment with an AI. Currently, ERα, PR, and HER-2/neu immunoreactivity in paraffin-embedded breast cancer tissue samples are used routinely as predictive markers for responsiveness to the anti-estrogen tamoxifen [8]–[10]. We believe that a similar approach could be adopted by clinicians for selection of breast cancer patients for AI therapy; however, the molecular predictors of response to AI treatment are currently being investigated [11].

**Figure 1:**
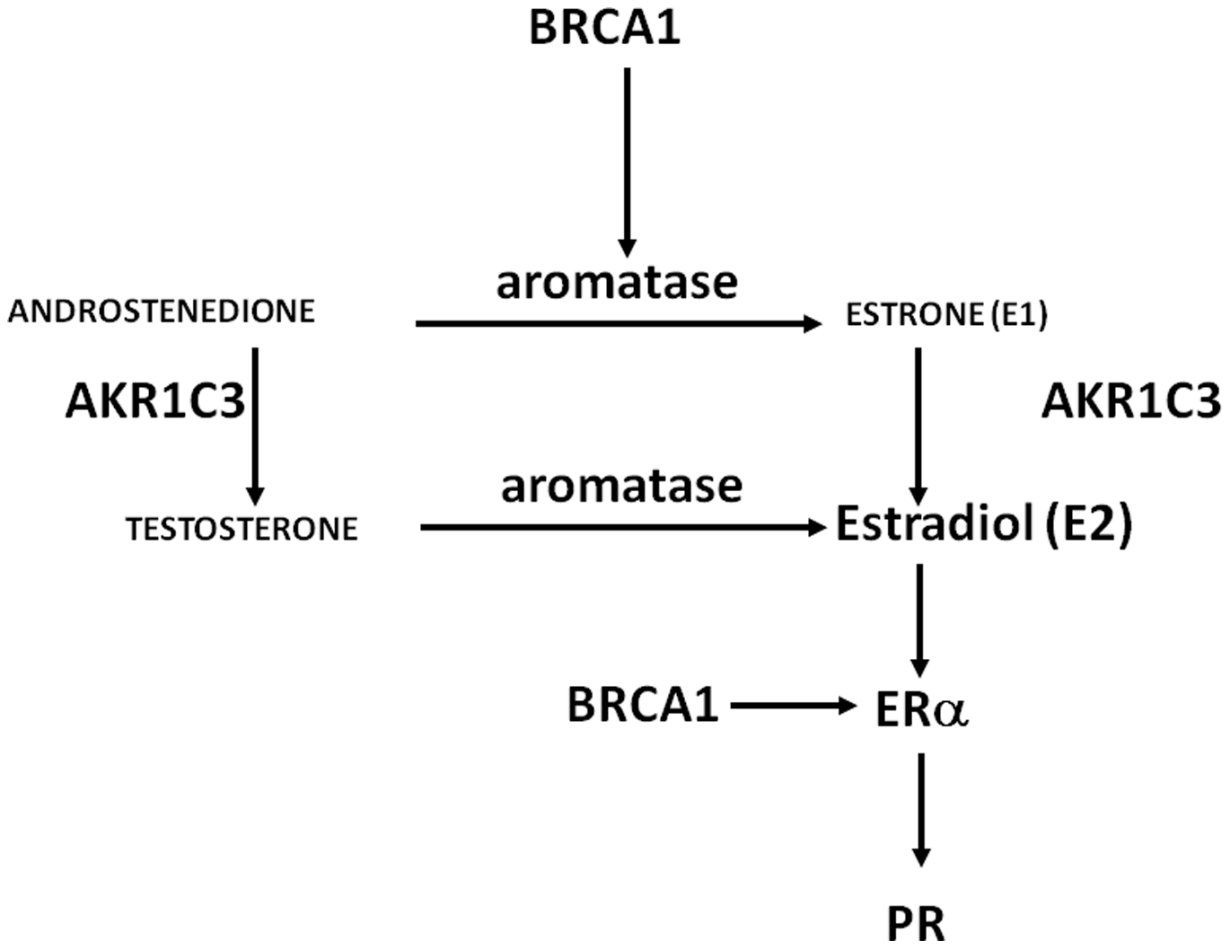
Estrogen-related genes in breast tumors. [Schematic of interplay between aromatase, BRCA1, AKR1C3, ERα, and PR.] Expression of these genes were chosen based on their relationship to estrogen in breast pathology. Aromatase is the key gene, whose product is responsible for estrogen formation. AKR1C3 encodes a key enzyme for the production of biologically active estrogen, estradiol, in breast cancer tissue. BRCA1 regulates aromatase and ER-alpha expression in the breast. PR is a prototype estradiol/ER-alpha responsive gene, although its absence may not always indicate a nonfunctional ER-alpha.

We assessed the intratumor protein and mRNA expression levels of 5 genes related to estrogen synthesis and function–genes encoding the steroidogenic enzymes AKR1C3 and aromatase; ERα; and 2 estradiol/ERα target genes, BRCA1 and PR (Figure 1) – and determined their ability to predict responsiveness to AI therapy in postmenopausal women with recurrent breast cancer who failed to respond to adjuvant tamoxifen therapy.

## Materials and Methods

### Patients

Written informed consent was obtained from each patient before participation. This study was approved by the Institutional Review Boards of the Royal Marsden Hospital, London, U.K. and Northwestern University. We studied primary malignant breast tumor samples from 112 postmenopausal women, defined as women aged ≥50 years who had not menstruated during the preceding 12 months, who had follicle stimulating hormone levels >40 IU/L, or who had undergone bilateral oophorectomy. All primary breast tumor samples had been excised between 1989–1995, frozen or fixed and embedded in paraffin, and stored at the breast cancer tissue bank of the Royal Marsden Hospital, London, U.K.

Of the 112 patients, 44 had received neoadjuvant therapy prior to surgery; of these, 26 patients received tamoxifen, 5 patients received chemotherapy, and 13 patients received a combination of tamoxifen and chemotherapy. All patients were treated with adjuvant tamoxifen. The mean duration of tamoxifen therapy was 42 months. All patients subsequently developed recurrent locally advanced or metastatic disease. Thereafter, the patients received either anastrozole (n = 101) or letrozole (n = 11). The hormone receptor status of the tumors had not been determined before administration of neoadjuvant or adjuvant therapy.

### Response Analysis

Computed tomography (CT) had been performed to measure the size of primary and metastatic lesions after patients had failed tamoxifen but before they started AI therapy (i.e., pretreatment), and also after 3 and 6 months of AI therapy. Measurable disease by CT scan included the primary tumor and metastatic disease. Osteolytic bone lesions, single metastatic lesions smaller than 0.5 cm, pleural effusions, or ascites were not considered measurable disease.

After initiation of AI therapy, patients had been assessed clinically every month for the first 3 months and then every 3 months until disease progression was detected. Responsiveness to AI therapy was measured by clinical benefit, which is defined as the complete response, partial response, or stable disease for at least 6 months of treatment [12]–[17]. Complete response was defined as no measurable tumor by CT scan. Partial response was defined as a reduction in tumor size ≥50% from pretreatment size. Stable disease was defined as <25% decrease or <25% increase in tumor size from pretreatment size. Progressive disease was defined as a ≥25% increase in tumor size from pretreatment size [17]. All patients continue to be followed to the present time, and all deaths recorded.

### Real-time RT-PCR

RNA was extracted from 112 frozen primary tumor tissue using the RNeasy Mini Kit (Qiagen, Valencia, CA) according to the manufacturer’s instructions. Tumor samples were fresh frozen and stored at −80°C prior to RNA extraction. The quantity and quality of the total RNA was analyzed using an Agilent 2100 Bioanalyzer RNA 6000 Nano LabChip kit (Agilent Technologies, Palo Alto, CA). Taqman real-time PCR primers and probe for aromatase mRNA were designed using ABI Primer Express Software 3.0 (Applied Biosystems, Carlsbad, CA). Reactions were carried out using the ABI Taqman assay system for aromatase mRNA. ABI power SYBR green PCR master mix was used for mRNAs encoding BRCA1, ERα, PR, or AKR1C3.

The following primer pairs were used for PCR [18]–[21]:

Aromatase primers: forward 5′-CACATCCTCAATACCAGGTCC-3′ and reverse 5′-CAGAGATCCAGACTCGCATG-3′

Aromatase probe: 5′ 6-FAM-CCCTCATCTCCCACGGCAGATTCC-TAMRA-3′

BRCA1 primers: forward, 5′-AAC CTG CTG ATG AAG TCA CAA-3′; reverse, 5′-TCA GAC ATT TAG GCA AGA CT-3′

ERα primers: forward 5′-AAGAGCTGCCAGGCCTGCC-3′ and reverse 5′-TTGGCAGCTCTCATGTCTCC-3′

PR primers: forward 5′-TCAGTGGGCAGATGCTGTATTT-3′ and reverse 5′-GCCACATGGTAAGGCATAATGA-3′

AKR1C3 primers: forward 5′-CAACCAGGTAGAATGTCATCCGTAT-3′ and reverse 5′-ACCCATCGTTTGTCTCGTTGA-3′

GADPH primers: forward 5′-GAAGGTGAAGGTCGGAGTC-3′ and reverse 5′-GAAGATGGTGATGGGATTTC-3′

GAPDH probe: 5′ 6-FAM CAAGCTTCCCGTTCTCAGCC-TAMRA 3′.

Real-time RT-PCR was performed on an Applied Biosystems Prism 7000 or 7900 HT sequence detection system (Applied Biosystems, Carlsbad, CA). Values for each gene were normalized to the expression levels of GAPDH. A known ERα and PR positive human proliferative endometrium was run as positive control for each run on the ABI 7900 apparatus. A dissociation curve was analyzed for each sample to ensure that a single amplification product was obtained.

### Immunohistochemistry (IHC)

Immunohistochemistry was performed on paraffin-embedded sections of 112 primary tumors. Immunostaining was carried out against ERα (Novocastra 6F11, Novocastra Laboratories, Ltd., Newcastle upon Tyne, UK) and PR (Novocastra 312) as described previously [4]. A cut-off of 1% positive cells was used to determine ER positivity and a cut-off of an H-score ≥20 was used to determine PR positivity [22]–[24]. Specifically, two pathologists independently evaluated the intensity of nuclear staining scored as 0, 1, 2, or 3 with 1 representing faint but distinct staining recognizable above negative controls, 3 representing the most intense staining seen, and 2 an intermediate. At least 200 cells were counted from each tumor. The ER or PR immunoreactivity was derived by multiplying staining intensity, percentage stained epithelial cells, and cellularity. To obtain an integer, the obtained value was multiplied by 100 to give a 0–300 score of ER or PR content. A known ER/PR-positive and ER/PR-negative specimen was incubated in parallel with the unknown sections with every run [22]–[25].

### Statistical Analysis

Fisher’s exact test was used to compare tumor characteristics between AI responsive categories. Contingency tables were constructed for protein (by IHC) and mRNA (by RT-PCR) expression levels to determine sensitivity, specificity, and positive predictive values for responsiveness to AI. Each marker was related to the response using logistic regression, Receiver Operating Characteristic (ROC) curves and area under the ROC (AUC) were employed; leave-one-out cross validation was performed by leaving one observation out, fitting a model with the remaining data points, then classifying the left out observation as a responder or non-responder. This was done for each observation so that the validation statistics were based on a sample size of 112 classified observations. Sensitivity and specificity was re-calculated based on a compilation of classified observations. Fisher’s exact test compared response; and the Wilcoxon rank sum test compared the median level of gene expression variables between patients who received neoadjuvant therapy and those who did not receive neoadjuvant therapy. McNemar’s test compared specificities between ERα-positive IHC versus ERα mRNA and between ERα- or PR-positive IHC versus ERα and PR mRNA at a level of identical sensitivity. Pearson correlation coefficients were calculated between the mRNA levels of estrogen-related genes in all tumors, in ERα protein-positive or -negative tumors, or in AI responders or non-responders. The statistical software SAS was used for the calculations (SAS Institute Inc., Cary, NC). Statistical significance was set at p<0.05.

## Results

### Tumor Characteristics and Response Rates

Tumor characteristics of the primary tumor samples are shown in Table 1. The overall AI response rate for the 112 patients was 51% (pre-test prevalence). We compared response rates between patients who received any neoadjuvant therapy to those who did not receive any neoadjuvant therapy. The AI response rate for 68 patients who did not receive neoadjuvant therapy (37/68 = 54.4%) was not significantly different from that in the neoadjuvant group of 44 patients (20/44 = 45.5%, p = 0.44). Given these findings, we performed a combined analysis including all 112 patients.

**Table 1:** Tumor characteristics.

	RESPONDERS (n = 57)	NON-RESPONDERS (n = 55)	P-value
**ER IHC positive**	96.5 (%)	78.2 (%)	0.004
**ER IHC negative**	3.5	21.8	
**PR IHC positive**	84.2 (%)	69.1 (%)	0.074
**PR IHC negative**	15.8	30.9	
**Grade 1**	3.5 (%)	5.4 (%)	0.96
**Grade 2**	40.4	40.0	
**Grade 3**	36.8	38.2	
**Grade not given**	19.3	16.4	
**1–10 mm**	3.5 (%)	9.1 (%)	0.17
**11–20 mm**	35.1	36.4	
**21–30 mm**	26.3	27.3	
**31–40 mm**	12.3	16.4	
**>40 mm**	12.2	10.9	
**Size not given**	10.5	0.0	
	**RESPONDERS With Neoadj (n = 20)**	**NON-RESPONDERS With Neoadj (n = 24)**	
**TAM only**	60.0 (%)	58.3 (%)	0.68
**Chemo only**	15.0	8.3	
**TAM + Chemo**	25.0	33.3	

### Response Rates by ERα and PR IHC Positivity

The overall AI response rate for ERα protein-positive patients and PR protein-positive patients was identical: 56%. By comparison, ERα protein-negative patients had a 14% response rate whereas PR protein-negative patients had a 35% response rate. ERα protein-positive status had a positive predictive value (PPV) of 56% and carried a sensitivity of 96% and a specificity of 22% (Table 2, Figure S1A). A positive PR protein status had a PPV of 56% with a sensitivity of 84% and specificity of 31% (Figure S1B). In current medical practice, patients with ERα or PR protein-positive tumors are treated with an AI. As shown in Table 2 and Figure S1C, the PPV of current medical practice was 54% with a sensitivity of 96% and a specificity of 16%.

**Table 2:** Comparison of Sensitivity and Specificity for IHC versus mRNA.

	TruePos	FalsePos	TrueNeg	FalseNeg	Sens	Spec
**ER IHC**	55	43	12	2	96%	22%
**ER or PR IHC**	55	46	9	2	96%	16%
**ER mRNA**	55	41	14	2	96%	25%
**ER or PR mRNA**	56	47	8	1	98%	15%
**ER/PR mRNA**	55	35	20	2	96%	36%

### Predictive Value of ERα and PR mRNA Expression for AI Responsiveness

A contingency table based on combined ERα and PR mRNA analysis resulted in comparable sensitivity to current medical practice (Table 2, Figure S2). ROC analysis revealed that ERα protein and mRNA have similar predictive value for AI response, in that specificity was 25% for mRNA and 22% for protein at 96% sensitivity. McNemar’s analysis revealed that there was no significant difference between these specificity levels when non-responders were analyzed for each marker (ERα mRNA versus protein, Table 2). Although there was no statistical difference between the sensitivities, ERα and PR mRNA combined analysis provided a statistically superior specificity (36%) compared with that of the current clinical practice (ERα or PR protein IHC, 16%) for prediction of response to AI treatment (p = 0.0013).

### AI Response Rates by mRNA Levels of Estrogen-related Genes

In addition to ERα and PR mRNA, we explored the prognostic value of aromatase, AKR1C3, and BRCA1 mRNA expression levels for predicting AI responsiveness. All of these genes play interconnected roles in breast cancer pathophysiology. The production of estradiol from androstenedione requires, in addition to aromatase, the reductive enzyme 17β-hydroxysteroid dehydrogenase to convert androstenedione to testosterone; in the breast, this enzyme is aldo-keto reductase (AKR1C3) [26], [27]. The aromatase enzyme catalyzes the key and last step in estrogen biosynthesis. Large amounts of aromatase mRNA, protein, and enzyme activity in breast tumors lead to the formation of substantial quantities of estrogen locally in this tissue that support tumorigenesis.

BRCA1 is well known for its role in maintaining genome stability by regulating homologous recombination of DNA damage [28]. BRCA1 has also been shown to interact with both ERα and the androgen receptor, and modify ERα signaling and estrogen target gene regulation [29]. Additionally, BRCA1 has been reported to regulate estrogen synthesis through transcriptional inhibition of aromatase [30], [31]. It is thought that heterozygosity status-reduced wild-type BRCA1 protein dosage (haploinsufficiency) and/or the presence of a mutant BRCA1 protein may affect BRCA1 functions and heighten the risk of cancer promoting mutations. The reduction of functional BRCA1 protein correlates with higher aromatase levels in 85% of BRCA1 mutation carriers [32].

We analyzed mRNA levels of estrogen related genes as potential predictors of response to an AI. First, we examined each individual mRNA as a potential predictor of response by ROC analysis in the entire sample of 112 tumors. Analysis of aromatase and AKRC13 mRNA did not reveal remarkable AUC values or statistically significant p-values. Three mRNA species demonstrated potentially meaningful AUCs, p-values, sensitivity, and specificity: ERα, PR, and BRCA1 (Table 3). The best predictor of AI responsiveness was the combination of ERα and PR mRNA levels, with a specificity of 36% and a sensitivity of 96% (p = 0.0009). Cross-validation by the leave-one-out method also indicated a sensitivity of 84% and specificity of 51%.

**Table 3:** ROC and logistic regression analysis of estrogen-related gene expression.

Intramural mRNAlevel	AUC(95% CI)	P-value	Specificity at96% sensitivity
**BRCA1**	0.618	0.048	5%
	(0.514, 0.722)		
**ERα**	0.691	0.0004	25%
	(0.594, 0.788)		
**PR**	0.679	0.0014	27%
	(0.580, 0.778)		
**BRCA1 + ERα**	0.692	0.0018	29%
	(0.595, 0.790)		
**BRCA1 + PR**	0.693	0.0031	25%
	(0.596, 0.790)		
**ERα + PR**	0.698	0.0009	36%
	(0.601, 0.795)		
**BRCA1 + ERα + PR**	0.704	0.0029	34%
	(0.608, 0.800)		

Wilcoxon rank sum test was performed to compare the median levels of gene expression variables between the 44 patients who received neoadjuvant therapy (e.g. TAM, chemotherapy, or both) and the 68 patients who did not receive neoadjuvant therapy. No differences in the mRNA levels of the estrogen-regulated genes were found between the two groups for aromatase (p = 0.56), AKRC13 (p = 0.59), ERα (p = 0.06), PR (p = 0.09), and BRCA1 (p = 0.21).

### Predictive Value of mRNA Analysis in ERα or PR IHC-negative Patients

Lastly, we examined the molecular profiles of ERα-negative or PR-negative tumors. Patients with these tumors would ordinarily be denied treatment with an AI, and we wanted to explore possible predictive molecular signatures for AI responsiveness in this patient group.

Out of 14 ERα IHC-negative breast tumors, only 2 showed AI responsiveness (Figure S1A), whereas out of 26 PR IHC-negative tumors, 9 showed AI responsiveness (Figure S1B). Seven of these 9 patients were ERα IHC positive. Although analysis of the ERα IHC-negative tumors showed various possible beneficial trends, a meaningful statistical analysis could not be performed due to small sample size (n = 14). However, non-significant trends were observed as evident by high AUCs with ERα, PR, or BRCA1 mRNA.

We proceeded with analyzing the PR IHC-negative group (n = 26). ERα mRNA in this PR-IHC negative group gave rise to an AUC of 0.824 (p = 0.02, Figure S3A), whereas BRCA1 mRNA was associated with an AUC of 0.817 (p = 0.033, Figure S3B) by logistic regression. A multivariate analysis combining ERα and BRCA1 mRNA levels in the PR-IHC negative group showed a strikingly high AUC of 0.902 (p = 0.043; Figure S3C). The specificity and PPV of combined ERα and BRCA1 mRNA for predicting AI response were both 100%. Thus, one can combine PR-IHC followed by ERα/BRCA1 mRNA analysis in the PR IHC-negative group. This algorithm increases the sensitivity of PR IHC from 84% to 96% without lowering its 31% specificity. Although this interesting exercise demonstrates a potential clinical use of mRNA analysis in receptor IHC-negative patients, it does not provide any advantage over the straight combined ERα/PR mRNA analysis (sensitivity 96%, specificity 36%, Figure S2) for predicting response to AI treatment.

### Correlations between Estrogen-related Gene Expression, ERα Protein Positivity, and AI Responsiveness

We demonstrated positive and significant correlations between BRCA1, ERα, PR, aromatase, and AKR1C3 mRNA expression in various subsets of breast tumors (Table S1). Surprisingly, ERα IHC-negative tumors showed higher correlation coefficients despite a strikingly lower sample size. There was no apparent difference between tumors from responders and non-responders.

## Discussion

In current medical practice, ER and PR status are critical factors in determining the use and predicting the benefit of adjuvant tamoxifen therapy [2]. Additionally, the management of breast cancer relies on the determination of various predictive as well as prognostic markers. A better prognosis is associated with the presence of molecular predictors of responsiveness to a particular targeted therapy. A number of studies have investigated both prognostic and predictive factors for endocrine treatment in ER-positive breast tumors and have reported various molecular markers. However, many of these studies were conducted in patients receiving tamoxifen [33]–[37]. Few studies have reported on the predictors of response to AI primary endocrine therapy in ER-positive breast tumors [38].

The superiority of AIs over tamoxifen in the treatment of postmenopausal breast cancer has been demonstrated by various trials such as the Arimidex, Tamoxifen, Alone or in Combination (ATAC) trial, BIG I-98, and TARGET [23], [38]–[43]. Pioneering studies of Buzdar, et al, have suggested a potential role of an aromatase inhibitor after tamoxifen therapy [44]. Our group found that ERα, PR, and BRCA1 mRNA levels could be used to better identify those postmenopausal breast cancer patients who will respond the best to AI therapy.

Although IHC assessment of ERα and PR protein expression is recommended by the American Society of Clinical Oncology to guide breast cancer treatment; there is still a widely held perception that IHC is unreliable and inaccurate in a good proportion of cases [24], [45]–[47]. Real time RT-PCR assays for ERα and PR mRNA expression have been proposed as a superior alternative to IHC. Numerous groups have shown a high degree of concordance between IHC and real-time RT-PCR, particularly with regard to assessment of ER expression [48]–[52]. Although AIs are currently used to treat millions of women with breast cancer, there is a lack of rigorous studies comparing the value of protein versus mRNA as predictors of AI responsiveness. In our study, we utilized receiver operator characteristic curves (ROC), area under the curve of ROC analysis as well as sensitivity, specificity, and positive predictive values to test the strength of association between clinical benefit from AI treatment as a second line therapy after failed TAM use with IHC and RT-PCR results. We found that ERα and PR mRNA levels measured by real-time RT-PCR was statistically superior to ERα or PR IHC for predicting AI response in cross-validated analyses. Additionally, a multivariate analysis of PR-negative patients, who would not normally be offered AI treatment, revealed that a specific subgroup may respond to AI therapy. Our treatment algorithm underscores the necessity to assess the combined ERα, PR, and BRCA1 mRNA expression patterns in all patients to better identify those who may benefit from AI therapy. In light of our small sample size, however, additional validation studies are needed to support a change in current practice standards.

Moreover, it is impractical to depend on frozen samples used in our study. In the future, being able to utilize RNA isolated from paraffin embedded samples will offer improved practicality over protein-IHC analysis obtained from frozen tissues because paraffin-embedded samples are more readily available than frozen tissues.

In summary, this study demonstrated a high concordance between IHC and real time RT-PCR for predicting responsiveness to an AI in patients who developed recurrent, advanced breast cancer after adjuvant tamoxifen therapy. Real time RT-PCR may offer a superior and more practical alternative to IHC for determining hormone receptor status, with improved specificity and PPV for predicting response to AI therapy, even in hormone receptor-negative (by IHC) patients. New and validated algorithms can augment the current standard practice of treating all hormone receptor-positive breast cancers by enabling better tailored treatment options based on the patient’s particular breast cancer molecular profile.

## Supporting Information

Figure S1 **AI response rates by ERα and PR IHC positivity.** (A) ERα IHC analysis. [Sensitivity, specificity, and positive-predictive values of ERα-IHC vs AI responsiveness].(B) PR IHC analysis. [Sensitivity, specificity, and positive-predictive values of PR-IHC vs AI responsiveness] (C) Treatment of ERα or PR IHC-positive tumors. [Sensitivity, specificity, and positive-predictive values of treated/untreated ERα or PR-positive tumors and AI responsiveness].
(PPTX)

Figure S2 **Predictive value of ERα and PR mRNA expression for AI.** (A) AI responsiveness by ERα and PR mRNA positivity. [Sensitivity, specificity, and positive-predictive values of ERα/PR-positive tumors determined by RT-PCR vs AI responsiveness].
(TIF)

Figure S3 **Logistic regression and ROC analysis of PR-negative tumors by ERα mRNA (A).** [Left panel shows Receiver Operating Characteristic curve relating ERα mRNA level to clinical benefit. Right panel is raw data at optimum cutpoint.] (B) Logistic regression and ROC analysis of PR-negative tumors by BRCA1 mRNA. [Left panel shows Receiver Operating Characteristic curve relating BRCA1 mRNA level to clinical benefit. Right panel is raw data at optimum cutpoint ]. (C) Treatment algorithm for PR IHC negative patients. [Left panel shows Receiver Operating Characteristic curve relating ERα and BRCA1 mRNA levels jointly to clinical benefit. Right panel is raw data at optimum cutpoint].
(PPTX)

Table S1 **Pearson correlation (p-value) analysis among estrogen-related gene expression measures for all patients and by ERα-IHC status and by AI responsiveness.**
(TIF)
